# Development of the Italian Version of the Near-Death Experience Scale

**DOI:** 10.3389/fnhum.2018.00045

**Published:** 2018-02-09

**Authors:** Francesca Pistoia, Giulia Mattiacci, Marco Sarà, Luca Padua, Claudio Macchi, Simona Sacco

**Affiliations:** ^1^Neurological Institute, Department of Biotechnological and Applied Clinical Sciences, University of L’Aquila, L’Aquila, Italy; ^2^Post-Coma Intensive and Rehabilitation Care Unit, San Raffaele Hospital, Cassino, Italy; ^3^Don Carlo Gnocchi Onlus Foundation, Milan, Italy; ^4^Department of Geriatrics, Neurosciences and Orthopaedics, Università Cattolica del Sacro Cuore, Rome, Italy; ^5^Don Carlo Gnocchi Foundation, Istituto di Ricovero e Cura a Carattere Scientifico, Florence, Italy; ^6^Department of Experimental and Clinical Medicine, University of Florence, Florence, Italy

**Keywords:** near-death experiences, coma, vegetative state, minimally conscious state, locked-in syndrome

## Abstract

Near-death experiences (NDEs) have been defined as any conscious perceptual experience occurring in individuals pronounced clinically dead or who came very close to physical death. They are frequently reported by patients surviving a critical injury and, intriguingly, they show common features across different populations. The tool traditionally used to assess NDEs is the NDE Scale, which is available in the original English version. The aim of this study was to develop the Italian version of the NDE Scale and to assess its reliability in a specific clinical setting. A process of translation of the original scale was performed in different stages in order to obtain a fully comprehensible and accurate Italian translation. Later, the scale was administered to a convenience sample of patients who had experienced a condition of coma and were, at the time of assessment, fully conscious and able to provide information as requested by the scale. Inter-rater and test–retest reliability, assessed by the weighted Cohen’s kappa (*K*_w_), were estimated. A convenience sample of 20 subjects [mean age ± standard deviation (SD) 51.6 ± 17.1, median time from injury 3.5 months, interquartile range (IQR) 2–10] was included in the study. Inter-rater [*K*_w_ 0.77 (95% CI 0.67–0.87)] and test–retest reliability [*K*_w_ 0.96 (95% CI 0.91–1.00)] showed good to excellent values for the total scores of the Italian NDE Scale and for subanalyses of each single cluster of the scale. An Italian Version of the NDE Scale is now available to investigate the frequency of NDE, the causes for NDE heterogeneity across different life-threatening conditions, and the possible neural mechanisms underlying NDE phenomenology.

## Introduction

The near-death experiences (NDEs) have been traditionally defined as “any conscious perceptual experience occurring in individuals pronounced clinically dead or who came very close to physical death” (Moody, 1975). More recently, NDEs have been better characterized as “a profound psychological event including transcendental and mystical elements, typically occurring to individuals close to death or in situations of intense physical or emotional danger” (Greyson, 2000). Almost one in four persons who survive a critical injury and between 4 and 9% of the general population experienced an NDE (Cant et al., 2012). Main features of the NDEs include out-of-body experiences, peaceful feelings, and transcendental or mystical experiences. Specifically, out-of-body experiences are characterized by a sensation of self-visualization from a position of height while transcendental or mystical experiences imply an apparent passage of the consciousness into a foreign dimension. The phenomenology of NDEs has been reported with sufficient consistency to consider them not as a cultural phenomenon but as a phenomenon with specific scientific features and underlying neural mechanisms. In fact, although no specific features have been universally described by all NDE experiencers, there are numerous cases of apparent correlations between specific NDEs features and circumstances in which the NDEs occur (Lake, 2017). All these observations prompted the diffusion of several studies investigating the frequency and relevance of NDEs, the causes for NDEs heterogeneity across different life-threatening conditions, and the possible neural mechanisms underlying NDEs phenomenology (Charland-Verville et al., 2014; Lake, 2017). Biological, psychological, and transpersonal models have been proposed to explain NDEs contents but there is still no consensus on a single unifying model (Lake, 2017).

The most widely used tool to assess NDEs as a unitary phenomenon is the Greyson NDE Scale (Greyson, 1983). However, it is available in the original English version only, which cannot be used in Italian patients because of intercultural differences and possible misunderstandings.

The objective of the present study was to develop and validate an Italian version of the NDE Scale in order to make it available for NDEs research.

## Materials and Methods

### Description of the NDE Scale

The NDE Scale has been derived by a previous questionnaire including 80 manifestations commonly described in the phenomenological literature of NDEs. Among these 80 manifestations, the 40 items most commonly reported were selected. A later analysis lead to the exclusion of further seven items because of their redundancy or ambiguity, with the remaining 33 items being reworded into questions with 3-point-scaled answers, to allow the scoring of each item as definitely present, questionable or atypical, and definitively absent (Greyson, 1983). This preliminary 33-item questionnaire was administered to 67 subjects in two times in order to assess internal consistency and test–retest reliability. The final NDE Scale resulting from this process included 16 questions grouped into four psychologically meaningful clusters: the cognitive cluster, the affective cluster, the paranormal cluster, and the transcendental cluster. For each item, the scores are arranged on an ordinal scale ranging from 0 to 2 (i.e., 0 = “not present,” 1 = “mildly or ambiguously present,” and 2 = “definitively present”) (Greyson, 1983; Lange et al., 2004). The final English scale is shown in **Table 1**.

**Table 1:** Original version of the NDE scale.

Component and question	Weighted response
**Cognitive**	
	2 = Everything seemed to be happening all at once
1. Did time seem to speed up?	1 = Time seemed to go faster than usual
	0 = Neither
	2 = Incredibly fast
2. Were your thoughts speeded up?	1 = Faster than usual
	0 = Neither
	2 = Past flashed before me, out of my control
3. Did scenes from your past come back to you?	1 = Remembered many past events
	0 = Neither
	2 = About the universe
4. Did you suddenly think to understand everything?	1 = About myself or others
	0 = Neither
**Affective**	
	2 = Incredible peace or pleasantness
5. Did you have a feeling of peace or pleasantness?	1 = Relief or calmness
	0 = Neither
	2 = Incredible joy
6. Did you have a feeling of joy?	1 = Happiness
	0 = Neither
	2 = United, one with the word
7. Did you feel a sense of harmony or unity with the universe?	1 = No longer in conflict with nature
	0 = Neither
	2 = Light clearly of mystical or other-worldly origin
8. Did you see or feel surrounded by a brilliant light?	1 = Unusually bright light
	0 = Neither
**Paranormal**	
	2 = Incredibly more so
9. Were your senses more vivid than usual?	1 = More so than usual
	0 = Neither
10. Did you seem to be aware of things going on elsewhere, as if by extrasensory perception?	2 = Yes, and facts later corroborated
	1 = Yes, but facts not yet corroborated
	0 = Neither
	2 = From the word’s future
11. Did scenes from the future come to you?	1 = From personal future
	0 = Neither
	2 = Clearly left the body and existed outside it
12. Did you feel separated from your physical body?	1 = Lost awareness of the body
	0 = Neither
**Transcendental**	
	2 = Clearly mystical or unearthly realm
13. Did you seem to enter some other, unearthly world?	1 = Unfamiliar, strange place
	0 = Neither
	2 = Definite being, or voice clearly of mystical or other-worldly origin
14. Did you seem to encounter a mystical being or presence?	1 = Unidentifiable voice
	0 = Neither
	2 = Saw them
15. Did you see deceased spirits or religious figures?	1 = Sensed their presence
	0 = Neither
	2 = A barrier I was not permitted to cross; or “sent back” to life involuntarily
16. Did you come to a border or point of no return?	1 = A conscious decision to “return” to life
	0 = Neither

This scale has been used in several studies investigating the prevalence of NDE in different populations. No studies have been carried out in the Italian population probably due to the lack of an Italian version of the scale.

### Development of the Italian Version of the NDE Scale

The following protocol was used to develop an Italian version of the NDE Scale:

(a) Three authors belonging to the research group completed three separate translations of the scale.
(b) A back translation of a selected version was made to check for any errors occurred during the original translation.
(c) The most accurate Italian translation was selected within a consensus meeting, with special attention being paid to the consistency of the new scale to the original one.
(d) A final back translation of the agreed Italian version was made as a further check.
The final Italian scale is shown in **Table 2**.

**Table 2:** Italian version of the NDE scale.

Componente e domanda	Risposta ponderata
**Cognitiva**	


	2 = Sembrava che le cose accadessero tutte in una volta


1. Il tempo sembrava scorrere più velocemente?	1 = Il tempo sembrava scorrere più velocemente del solito


	0 = Nessuna delle due


	2 = In maniera incredibilmente veloce


2. I tuoi pensieri fluivano più velocemente?	1 = Più velocemente del solito


	0 = Nessuna delle due


	2 = Il passato si è palesato davanti a me, fuori dal mio controllo


3. Ti sono tornate in mente scene del tuo passato?	1 = Ho ricordato molti eventi passati


	0 = Nessuna delle due


	2 = Sì, a proposito dell’Universo


4. Ti è sembrato di comprendere improvvisamente ogni cosa?	1 = Sì, riguardo me stesso e gli altri


	0 = Nessuna delle due


**Affettiva**	


	2 = Un’incredibile sensazione di pace o appagamento


5. Hai provato una sensazione di pace o appagamento?	1 = Una sensazione di sollievo o quiete


	0 = Nessuna delle due


	2 = Una sensazione di gioia incredibile


6. Hai provato una sensazione di gioia?	1 = Felicità


	0 = Nessuna delle due


	2 = Mi sono sentito una cosa sola con il mondo


7. Hai provato un senso di armonia o unità con l’universo?	1 = Non mi sono più sentito in conflitto con la natura


	0 = Nessuna delle due


	2 = Una luce di origine chiaramente mistica o di un altro mondo


8. Hai visto o ti sei sentito avvolto da una luce brillante?	1 = Una luce insolitamente brillante


	0 = Nessuna delle due


**Paranormale**	


	2 = Incredibilmente più vividi


9. I tuoi sensi sembravano essere più vividi del solito?	1 = Più vividi del solito


	0 = Nessuna delle due


10. Ti è sembrato di essere consapevole di cose che stavano accadendo altrove, come in un’esperienza extra-sensoriale?	2 = Sì, e, successivamente, i fatti lo hanno confermato

	1 = Sì, ma i fatti non lo hanno confermato

	0 = Nessuna delle due


	2 = Sì, relative al futuro del mondo


11. Ti sono apparse scene dal futuro?	1 = Sì, relative al mio personale futuro


	0 = Nessuna delle due


	2 = Sì, ho chiaramente abbandonato il corpo e ho percepito di esistere al di fuori di esso


12. Hai avvertito una sensazione di separazione dal tuo corpo fisico?	1 = Ho perso la consapevolezza del mio corpo


	0 = Nessuna delle due


**Transcendentale**	


	2 = Sì, in un regno chiaramente mistico o soprannaturale


13. Ti è sembrato di entrare in un mondo soprannaturale?	1 = Sì, in un luogo estraneo, non familiare


	0 = Nessuna delle due


	2 = Sì, con un essere definito o una voce di origine chiaramente soprannaturale o mistica


14. Ti è sembrato di entrare in contatto con un essere o una presenza mistica?	1 = Sì, con una voce non identificabile


	0 = Nessuna delle due


	2 = Li ho visti


15. Hai visto lo spirito di persone decedute o figure religiose?	1 = Ho avvertito la loro presenza


	0 = Nessuna delle due


	2 = Sì, a una barriera che non mi era permesso di oltrepassare/o sono stato rimandato indietro in vita involontariamente


16. Sei arrivato a un confine o ad un punto di non ritorno?	1 = Ho preso la decisione conscia di ritornare alla vita


	0 = Nessuna delle due

### Assessment of Reliability of the Italian Version of the NDE Scale

A convenience sample of 20 patients was included in this study. Patients were recruited from Acute Inpatient Rehabilitation Units of the San Raffaele Hospital, Cassino, Italy and Don Gnocchi Foundation, Firenze, Italy. Inclusion criteria were the following: age > 18 years; Italian nationality; experience of a condition of coma in the last 5 years as a result of a severe brain injury; and good cognitive status at the time of the assessment as established by the Level of Cognitive Functioning Scale (Level 8) (Gouvier et al., 1987). Patients with previous/concurrent severe neurological or psychiatric diseases or taking Central Nervous System acting drugs at the time of the interview were excluded from the study. Diagnostic reliability across raters (inter-rater reliability) and ratings (intra-rater reliability) was investigated as follows: the Italian Version was administered to all the included patients by two health care professionals in the same day. The two raters independently assessed each patient and recorded both total scores and single-item subscores. Each rater was blinded with respect to the information collected by the other rater. After 2 weeks, a second evaluation by the first rater was made in order to estimate test–retest reliability. In the case of patients with locked-in syndrome (LIS), in whom the only movements preserved are blinking and vertical eye movements, the clinical interview was made by means of an eye-coded channel as usually it occurs in these patients. Main anagraphical and clinical data of all the patients were also collected. This study was approved by the Internal Review Board of the University of L’Aquila (Approval number 01/2018) and carried out in accordance with its recommendations.

### Statistical Analysis

Descriptive statistics were reported as mean ± standard deviation (SD) or median with interquartile range (IQR) for quantitative variables and as counts and proportions (%) for categorical variables. Inter-rater and test–retest reliability were assessed by the weighted Cohen’s kappa (*K*). κ-values were interpreted as follows: >0.80 excellent agreement, 0.61–0.80 good agreement, 0.41–0.60 moderate agreement, 0.21–0.40 fair agreement, and <0.21 poor agreement (Altman, 1991). Data analysis was performed using the IBM SPSS Statistics 20.0. The level of significance was α = 0.05.

## Results

All the included patients [14 males and 6 females, mean age ±*SD* 51.6 ± 17.1, median time from injury 3.5 months, interquartile range (IQR) 2–10] experienced a condition of coma in the acute phase of their disease. When entering the rehabilitation ward most of the patients were in a condition of minimally conscious state (MCS; *n* = 15; 75%). The remainder patients were fully conscious (*n* = 4; 20%) or showed a condition of emergence from MCS (EMCS; *n* = 1; 5%). Among patients with a normal consciousness at admission in the Acute Inpatient Rehabilitation Unit, one showed a condition of LIS. The primitive disease, being responsible for the loss of consciousness in the acute phase, was mainly represented by stroke (*n* = 12), followed by traumatic brain injury (*n* = 7) and infectious disease (*n* = 1). At the time of the interview all the included patients were fully conscious and cognitively preserved as established by the inclusion criteria.

The mean score of the NDE Scale in our sample was 3 ± 4.0 (range 0–13). The experiences most frequently reported by patients were those belonging to the cognitive component, being mainly represented by phenomena of time distortion (*n* = 5; 25%), life review (*n* = 5; 25%), and thought acceleration (*n* = 4; 20%). Experiences belonging to the transcendental component were also frequently recorded, especially those involving the view of deceased spirits or religious figures (*n* = 4; 20%). Only three patients reported a minimum score of 7 or higher, which is the cut-off of the original NDE Scale to establish the presence of a NDE as a unitary phenomenon (Greyson, 1983).

Inter-rater [*K*_w_ 0.77 (95% CI 0.67–0.87)] and test–retest reliability [*K*_w_ 0.96 (95% CI 0.91–1.00)] showed good to excellent values for the total scores of the Italian NDE Scale and for subanalyses of each single cluster of the scale as shown in **Table 3**.

**Table 3:** Inter-rater and test–retest reliability as estimated by Cohen’s kappa (*K*) values for total scores and individual clusters of the scale.

	Inter-rater reliability κ (95% CI)	Test–retest reliability κ (95% CI)
Cognitive cluster	0.80 (0.64–0.96)	0.92 (0.80–1.0)
Affective cluster	0.65 (0.28–1.0)	1.0 (-)
Paranormal cluster	0.78 (0.59–0.97)	1.0 (-)
Transcendental cluster	0.78 (0.57–0.99)	0.94 (0.83–1.0)
Total	0.77 (0.67–0.87)	0.96 (0.91–1.0)

## Discussion

Our findings provide support for the use of the Italian version of the NDE Scale in clinical research. To the best of our knowledge, there are no studies investigating the prevalence of NDEs in the Italian population. This is probably due to the lack of an Italian version of the scale, which is traditionally used to assess NDEs. In this study we provided a reliable Italian Version of the NDE scale showing an excellent inter-rater and intra-rater agreement. This tool may be used to investigate NDEs in subjects who experienced a condition close to death (real NDE) or non-life-threatening events without any brain damage (NDE-like experiences) (Charland-Verville et al., 2014). Patients with a past history of coma are the ideal candidates to investigate real NDE, when residual cognitive dysfunctions do not interfere with the clinical interview (Teasdale and Jennett, 1974; Plum and Posner, 1983). They include patients who directly recovered consciousness after a transitory phase of coma or patients who entered intermediates states, such as VS or MCS, before recovering consciousness (Giacino et al., 2002; Pistoia and Sarà, 2012; Pistoia et al., 2013; Bayne et al., 2017). Patients with LIS, showing a condition of coma in the acute stage of their disease, are also frequently asked to report about NDE (Charland-Verville et al., 2015). Patients with LIS, although being traditionally described as cognitively intact, often show a series of non-motor symptoms that can be interpreted in the framework of an embodiment disorder (Sacco et al., 2008; Babiloni et al., 2010; Pistoia et al., 2010, 2017). Intriguingly, some of these symptoms seem to share common features and similar underlying neurophysiological mechanisms with some NDEs phenomena, especially those involving out-of-body perceptions and emotional engagement. This makes such patients deserving of special attention to these symptoms, whether they occur near death or in the later stage of their diseases, in order to gain a better understanding of NDEs and to select rehabilitative approaches tailored to the patients’ specific characteristics (Conson et al., 2008, 2009, 2010).

Previous research on NDEs is extremely heterogeneous, depending on the clinical characteristics of the targeted research population. NDEs mainly include pleasant feelings such as peacefulness, painlessness, and joy. Less pleasant feelings have been reported in a minority of near-death experiencers, including patients with LIS (Charland-Verville et al., 2015). Brain injury-related mechanisms, which can influence the development of NDEs in coma survivors, include anoxic brain damage, hypoxia, hypercapnia, abnormal temporal lobe dysfunctions, and administration of sedatives (Charland-Verville et al., 2014). An interesting study recently investigated differences in NDEs characteristics depending on the brain injury etiology but it failed to detect any differences in intensity or contents across different brain diseases (Charland-Verville et al., 2014). However, it should be stressed that NDEs have been described non only in life-threatening events directly involving the brain but also in multiple other conditions including cardiac arrest, traumatic circumstances, and conditions of altered mental status under the influence of potentially psychoactive medications (Curran, 2000; Parnia et al., 2007). Moreover, a wide spectrum of NDE-like experiences after non-life-threatening events, not associated with real closeness to death or coma, has been described (Charland-Verville et al., 2014). This suggests that other mechanisms may be engaged in the development of NDE phenomenology (Facco and Agrillo, 2012).

The strengths of our study included the use of a standardized protocol in the process of translation and in the assessment of inter-rater and test–retest reliability, and the selection of a target population who experienced real life-threatening conditions as a result of a wide spectrum of brain injuries. Moreover, we included patients in whom consciousness recovery occurred in different times and modalities: some patients directly moved from coma to a condition of full consciousness while others experienced intermediate states such as VS and MCS.

A limitation of our study lies with the inclusion of a small sample, which avoided us to perform subgroup analyses in order to investigate NDEs consistency or heterogeneity across coma of different aetiologies and duration. However, this was beyond the aim of this study, which was mainly intended to provide a screening instrument to identify NDEs among unselected patients and to expand the research in this field.

The frequency of NDEs phenomena in our sample was low, with only three patients having shown a minimum score of 7 or higher, which is the cut-off of the original NDE Scale to establish the presence of a NDE as a unitary phenomenon. This finding is in line with previous incidence reports in post head injury patients (Hou et al., 2013). Further studies are necessary in order to better assess the frequency, intensity, and heterogeneity of NDEs across different diseases and conditions as well as their impact on belief systems and emotions of patients experiencing them. Finally, studies based on larger samples of patients coming from different countries will also allow to identify any additional set of phenomena, not being clearly detected by the present tool, and to further expand the field of research across different cultural settings.

## Author Contributions

All authors (FP, GM, MS, LP, CM, and SS) provided their substantial contributions to the conception of the work, the acquisition, analysis, and interpretation of data and to the draft writing.

## Conflict of Interest Statement

The authors declare that the research was conducted in the absence of any commercial or financial relationships that could be construed as a potential conflict of interest.

## References

[B1] Altman D. G. (1991). *Practical Statistics for Medical Research.* London: Chapman and Hall.

[B2] Babiloni C. Pistoia F. Sarà M. Vecchio F. Buffo P. Conson M. (2010). Resting state eyes-closed cortical rhythms in patients with locked-in-syndrome: an EEG study. *Clin. Neurophysiol.* 121 1816–1824. 10.1016/j.clinph.2010.04.027 20541461

[B3] Bayne T. Hohwy J. Owen A. M. (2017). Reforming the taxonomy in disorders of consciousness. *Ann. Neurol.* 82 866–872. 10.1002/ana.25088 29091304

[B4] Cant R. Cooper S. Chung C. O’Connor M. (2012). The divided self: near death experiences of resuscitated patients–a review of literature. *Int. Emerg. Nurs.* 20 88–93. 10.1016/j.ienj.2011.05.005 22483004

[B5] Charland-Verville V. Jourdan J. P. Thonnard M. Ledoux D. Donneau A. F. Quertemont E. (2014). Near-death experiences in non-life-threatening events and coma of different etiologies. *Front. Hum. Neurosci.* 8:203. 10.3389/fnhum.2014.00203 24904345 PMC4034153

[B6] Charland-Verville V. Lugo Z. Jourdan J. P. Donneau A. F. Laureys S. (2015). Near-death experiences in patients with locked-in syndrome: not always a blissful journey. *Conscious. Cogn.* 34 28–32. 10.1016/j.concog.2015.03.011 25837796

[B7] Conson M. Pistoia F. Sarà M. Grossi D. Trojano L. (2010). Recognition and mental manipulation of body parts dissociate in locked-in syndrome. *Brain Cogn.* 73 189–193. 10.1016/j.bandc.2010.05.001 20537784

[B8] Conson M. Sacco S. Sarà M. Pistoia F. Grossi D. Trojano L. (2008). Selective motor imagery defect in patients with locked-in syndrome. *Neuropsychologia* 46 2622–2628. 10.1016/j.neuropsychologia.2008.04.015 18533201

[B9] Conson M. Sarà M. Pistoia F. Trojano L. (2009). Action observation improves motor imagery: specific interactions between simulative processes. *Exp. Brain Res.* 199 71–81. 10.1007/s00221-009-1974-3 19690843

[B10] Curran H. V. (2000). “Psychopharmacological perspectives on memory,” in *The Oxford Handbook of Memory*, eds Tulving E. Craig F. I. M. (New York, NY: Oxford University Press), 539–554.

[B11] Facco E. Agrillo C. (2012). Near-death-like experiences without life-threatening conditions or brain disorders: a hypothesis from a case report. *Front. Psychol.* 3:490. 10.3389/fpsyg.2012.00490 23162522 PMC3498963

[B12] Giacino J. T. Ashwal S. Childs N. Cranford R. Jennett B. Katz D. I. (2002). The minimally conscious state: definition and diagnostic criteria. *Neurology* 58 349–353. 10.1212/WNL.58.3.349 11839831

[B13] Gouvier W. D. Blanton P. D. LaPorte K. K. Nepomuceno C. (1987). Reliability and validity of the disability rating scale and the levels of cognitive functioning scale in monitoring recovery from severe head injury. *Arch. Phys. Med. Rehabil.* 68 94–97. 10.1097/00001199-198712000-00015 3813863

[B14] Greyson B. (1983). The near-death experience scale. Construction, reliability, and validity. *J. Nerv. Ment. Dis.* 171 369–375. 10.1097/00005053-198306000-00007 6854303

[B15] Greyson B. (2000). “Near-death experiences,” in *Varieties of Anomalous Experiences: Examining the Scientific Evidence*, eds Cardena E. Lynn S. Krippner S. (Washigton, DC: American Psychological Association), 315–352.

[B16] Hou Y. Huang Q. Prakash R. Chaudhury S. (2013). Infrequent near death experiences in severe brain injury survivors - A quantitative and qualitative study. *Ann. Indian Acad. Neurol.* 16 75–81. 10.4103/0972-2327.107715 23661968 PMC3644787

[B17] Lake J. (2017). The near-death experience: a testable neural model. *Psychol. Conscious. Theory Res. Pract.* 4 115–134. 10.1037/cns0000099

[B18] Lange R. Greyson B. Houran J. (2004). A Rasch scaling validation of a ‘core’ near-death experience. *Br. J. Psychol.* 95(Pt 2), 161–177. 10.1348/000712604773952403 15142300

[B19] Moody R. A. (1975). *Life After Life.* New York, NY: Bantam Press.

[B20] Parnia S. Spearpoint K. Fenwick P. B. (2007). Near death experiences, cognitive function and psychological outcomes of surviving cardiac arrest. *Resuscitation* 74 215–221. 10.1016/j.resuscitation.2007.01.020 17416449

[B21] Pistoia F. Carolei A. Sacco S. Sarà M. (2017). Commentary: embodied medicine: mens sana in corpore virtuale sano. *Front. Hum. Neurosci.* 11:381. 10.3389/fnhum.2017.00381 28785213 PMC5519604

[B22] Pistoia F. Conson M. Trojano L. Grossi D. Ponari M. Colonnese C. (2010). Impaired conscious recognition of negative facial expressions in patients with locked-in syndrome. *J. Neurosci.* 30 7838–7844. 10.1523/JNEUROSCI.6300-09.2010 20534832 PMC6632697

[B23] Pistoia F. Sarà M. (2012). Is there a cartesian renaissance of the mind or is it time for a new taxonomy for low responsive states? *J. Neurotrauma* 29 2328–2331. 10.1089/neu.2009.1257 21488720

[B24] Pistoia F. Sarà M. Sacco S. Carolei A. (2013). Vegetative states and minimally conscious states revisited: a Russian doll approach. *Brain Inj.* 27 1330–1331. 10.3109/02699052.2013.809554 23924313

[B25] Plum F. Posner J. B. (1983). *The Diagnosis of Stupor and Coma.* Philadelphia, PA: F. A. Davis.

[B26] Sacco S. Sarà M. Pistoia F. Conson M. Albertini G. Carolei A. (2008). Management of pathologic laughter and crying in patients with locked-in syndrome: a report of 4 cases. *Arch. Phys. Med. Rehabil.* 89 775–778. 10.1016/j.apmr.2007.09.032 18374012

[B27] Teasdale G. Jennett B. (1974). Assessment of coma and impaired consciousness. A practical scale. *Lancet* 2 81–84. 10.1016/S0140-6736(74)91639-0 4136544

